# Detection of Residual Pesticides in Foods

**DOI:** 10.3390/foods10051113

**Published:** 2021-05-18

**Authors:** Roberto Romero-González

**Affiliations:** Research Group “Analytical Chemistry of Contaminants”, Department of Chemistry and Physics, Research Centre for Mediterranean Intensive Agrosystems and Agrifood Biotechnology (CIAIMBITAL), Agrifood Campus of International Excellence (ceiA3), University of Almeria, E-04120 Almeria, Spain; rromero@ual.es; Tel.: +34-950214278

Pesticides are used worldwide. Despite the fact that organic farming is increasingly popular, pesticides are still widely applied in many countries under different pesticide regulations and monitoring programs. To provide reliable results, robust and sensitive analytical methodologies based on chromatographic techniques coupled to mass spectrometry (MS) are widely used. Another important issue is the application of generic extraction methods, allowing for the extraction of pesticides with different physico-chemical properties. The aim of the Special Issue “Detection of Residual Pesticides in Foods” was to gather original research papers focused on the development, validation and application of analytical methods, based on hyphenated techniques (chromatography-MS), in pesticide residue analyses, bearing in mind that they combine the separation capacity of the chromatographic techniques with the identification power of MS. This Special Issue is comprised of nine valuable scientific contributions, covering different topics related to pesticide residue monitoring.

One of the main aims in the study of pesticide residue is increasing the scope of the analysis; therefore, multiresidue methods have been proposed. Almeida et al. [1] developed a methodology for the determination of 168 pesticide residues in honey by liquid chromatography-tandem mass spectrometry (LC-MS/MS) (127 compounds) and gas chromatography (GC)-MS/MS (41 pesticides), performing an extraction procedure based on the QuEChERS approach. Suitable validation parameters were achieved, and the method was applied to more than 30 honey samples; it was observed that carbendazim (20 samples), thiabendazole (20 samples), azoxystrobin (15 samples), chlorpyrifos (12 samples) and imidacloprid (12 samples) were the compounds most frequently detected, indicating that pesticide residues should be monitored in this sensitive matrix. In addition to the number of compounds, sample throughput is another key point to be considered when analytical methods are being developed. Grande-Martinez et al. [2] developed a 7 min multifamily residue method for the simultaneous quantification and confirmation of 8 phytohormones and 27 acidic herbicides in fruit and vegetables using ultra high-performance liquid chromatography (UHPLC) coupled to MS/MS. The method, which was validated according to SANTE 12682/2019, was also accredited (UNE-EN-ISO/IEC 17025:2017). Although sample treatment was based on the QuEChERS approach, because of the special characteristics of these kinds of compounds, a previous step of alkaline hydrolysis was needed. The proposed method was applied to the analysis of more than 450 samples of cucumber, orange, tomato, watermelon, and zucchini, and several compounds, such as 2,4-dichlorophenoxyacetic acid (2,4-D), 4-(3-indolyl)butyric acid (IBA), dichlorprop (2,4-DP), 2-methyl-4-chlorophenoxy acetic acid (MCPA), and triclopyr were detected, but at concentrations below the maximum residue level (MRL) regulated by the European Union (EU). Despite to the fact that QuEChERS can be widely used for the extraction of pesticide residues from vegetables, some compounds, such as sulfonylurea herbicides, are poorly recovered, and different modifications should be applied. Thus, dispersive solid phase extraction (d-SPE) sorbents should be carefully evaluated, considering that C18 is the most suitable sorbent for the determination of these compounds in strawberries by LC-MS/MS, achieving suitable precision and recovery [3].

The determination of highly polar pesticides is still a problem, and laboratories are looking for a pluri-residue method that encompasses the largest number of polar pesticides. In this sense, different stationary phases have been tested, and Manzano-Sánchez et al. [4] observed that the stationary phase Torus DEA provided the best separation of ethephon, 2-hydroxyethylphosphonic acid (HEPA), fosetyl aluminum, glyphosate, aminomethylphosphonic acid (AMPA), N-acetyl-glyphosate, and N-acetyl-AMPA. Previous LC separation, QuPPe method was used for the extraction of the targeted compounds, but slight modifications were necessary depending on the tested matrix (tomato, orange, aubergine, grape). For the detection of the compounds, a high-resolution single mass spectrometer, such as an Exactive-Orbitrap analyzer, provided reliable identification, considering that in addition to the characteristic ion, at least two fragments were monitored per compound.

Nowadays, it is also relevant to understand the dissipation of parent compounds to determine their persistence in different matrices. Thus, in a first study, aryloxyphenoxy-propionates and cyclohexanediones herbicide dissipation in vegetables was evaluated using LC-MS/MS after QuEChERS extraction. Non-linear models were applied, and it was observed that aryloxyphenoxy-propionates can contaminate vegetables with a short growing season and vegetables treated with fluazifop may not be suitable for baby food; however, propaquizafop and cycloxidim were found to be prospective herbicides for non-residual (baby food) vegetable production [5]. In a second study related to this topic, the degradation of 32 active substances (15 fungicides and 17 insecticides) was analyzed in different matrices, such as iceberg lettuce, onion, leek, carrot, and parsley. A first-order kinetic model was used, allowing the determination of an action pre-harvest interval based on an action threshold of 0.01 mg kg^−1^ to produce vegetables intended for zero-residue production. It was observed that the highest amount of pesticide residues was found in carrot and parsley leaves, and pesticide dissipation was generally slow. Lower amounts were found in leeks and lettuce. The authors indicate that it seems feasible to apply reduced pesticide amounts to stay below unwanted residue levels [6].

Related to this issue, in addition to the determination of parent compounds, metabolites are also being monitored in food and environmental matrices, bearing in mind that they can be more toxic or persistent than parent compounds. In a research article focused on this topic, three pyrethroid metabolites—3-phenoxybenzoic acid (3-PBA), 4-fluoro-3-phenoxybenzoic acid (4-F-3-PBA), and cis-3-(2-chloro-3,3,3-trifluoroprop-1-en-1-yl)-2,2-dimethylcyclopropanecarboxylic acid (TFA)—were determined in tea by applying QuEChERS and UHPLC-MS/MS. Because of the complexity of the matrix, a clean-up step based on d-SPE was necessary, and a mixture of graphitized carbon black (GCB), florisil, and C18 was needed. Lower limits of quantification (LOQs) were achieved, from 2 to 10 µg kg^−1^, and the validated method was applied to different types of tea, detecting 3-PBA and TFA in two samples [7].

Finally, monitoring activities are also important to achieve a full overview of the presence of these contaminants in foods of different origins. The study carried out by Panseri et al. [8] investigated the presence of contaminant residues (persistent organic pollutants (POPs) and pesticides, including glyphosate and metabolites) in organic honey samples from different production areas to confirm their incidence and possible impact on the food safety traits of organic production. Whereas GC-MS/MS was used for the determination of non-polar compounds, ionic chromatography coupled to Q-Exactive Orbitrap was used for glyphosate, glufosinate, and AMPA monitoring. Traces of organochlorine and organophosphate pesticides were detected in honey samples, in addition to other persistent organic pollutants. According to these results, it would seem mandatory to intensify the safety monitoring of this foodstuff and to keep improving good beekeeping practices, as suggested by the EU framework. In addition to vegetables, pesticides can also be detected in other matrices, such as meat. In the study performed by Kartalovic et al. [9], 19 organochlorine pesticides (OCPs) and other compounds were monitored in smoked meat by GC-MS; α-HCH, lindane, PCB 28, PCB 52, and PCB 153 were detected in the analyzed samples, highlighting that the concentrations of OCPs and PCBs were not significantly affected by product type and by conditions of production.

In summary, this Special Issue, Detection of Residual Pesticides in Foods, highlights that even though much work has been done in pesticide residue analysis, there are still some gaps that should be covered (e.g., dissipation studies, metabolites, determination of orphan compounds). For this purpose, hyphenated techniques are valuable tools to achieve sound results and improve the information related to the presence of pesticides in foods of different origins.
